# Supplementary figures and images for: Genomic organization and expression profiles of nitrogen assimilation genes in Glycine max
Source: PeerJ. 2024 Jun 24;12:e17590. doi: 10.7717/peerj.17590 (PMC11210457; doi:10.7717/peerj.17590)

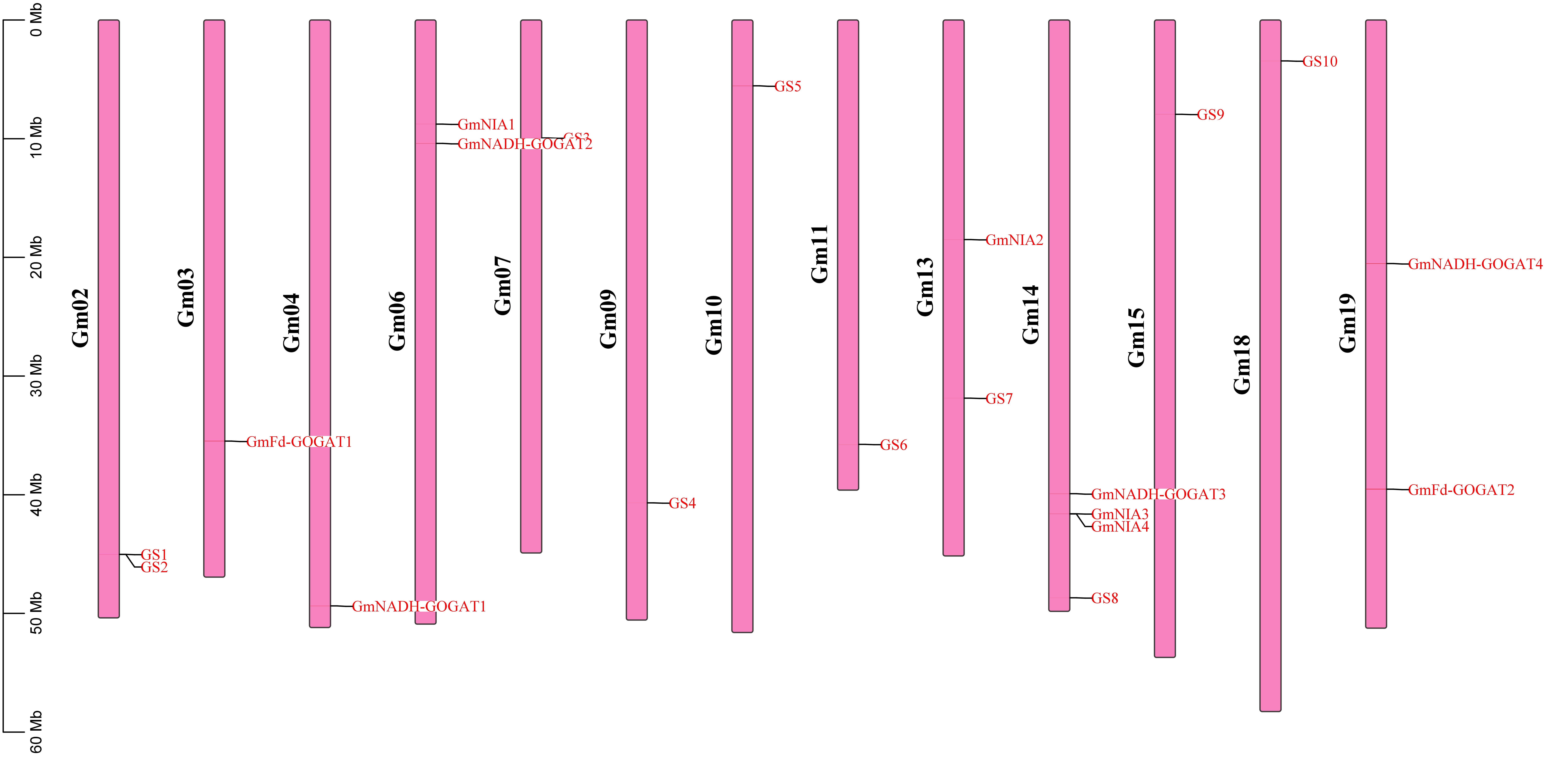

Supplement: Supplemental Information 1 — The scale represented the length of chromosomes, whereas the pink bars indicated chromosomes. The chromosome number is displayed on the left side of each pink bar. [file peerj-12-17590-s001.jpg]
